# Maternal exposure to di-n-butyl phthalate (DBP) induces renal fibrosis in adult rat offspring

**DOI:** 10.18632/oncotarget.16088

**Published:** 2017-03-10

**Authors:** Yi-Ping Zhu, Lei Chen, Xing-jie Wang, Qi-Heng Jiang, Xiao-Yu Bei, Wen-Lan Sun, Shu-Jie Xia, Jun-Tao Jiang

**Affiliations:** ^1^ Department of Urology, Shanghai General Hospital, Shanghai Jiao Tong University School of Medicine, Shanghai 200080, China; ^2^ Department of Geriatrics, Shanghai General Hospital, Shanghai Jiao Tong University School of Medicine, Shanghai 200080, China; ^3^ Medical Services Section, Shanghai General Hospital, Shanghai Jiao Tong University School of Medicine, Shanghai 200080, China

**Keywords:** renal fibrosis, kidney dysplasia, di-n-butyl phthalate (DBP), oxidative stress, environmental endocrine-disrupting compounds (EEDs)

## Abstract

This study was to determine the impact of maternal exposure to di-n-butyl phthalate (DBP) on renal development and fibrosis in adult offspring. Pregnant rats received DBP at a dose of 850 mg/kg BW/day by oral perfusion during gestational days 14–18. In DBP exposed newborn offspring, gross observation and histopathological examination revealed the dysplasia of kidney. The expression of genes related to renal development was also changed. In DBP exposed adult offspring, histopathological examination and Masson's trichrome staining revealed the pathological changes of renal fibrosis. Furthermore, higher expression levels of transforming growth factor- β (TGF-β) and alpha-smooth muscle actin (α-SMA) were also detected. *In vitro* studies reveal that DBP promoted the activation of NRK49F cells and G2/M arrest in NRK52E cells at a sublethal dose. The effect of DBP on these cell lines was linked to the generation of oxidative stress. In addition, DBP induced oxidative stress in both renal fibroblasts and tubular epithelial cells, whereas vitamin C ameliorated the changes caused by DBP. In conclusion, our results showed that prenatal exposure to DBP may generate oxidative stress in both renal fibroblasts and tubular epithelial cells, leading to kidney dysplasia and renal fibrosis.

## INTRODUCTION

It is estimated that 8–16% of adults in the world have chronic kidney disease (CKD), which is characterized by relentless deposition of extracellular matrix and progressive tubulointerstitial fibrosis (TIF). TIF eventually leads to end-stage renal disease, which seriously affects the quality of life and results in heavy financial burden [2–4]. Therefore, exploring of the pathologic mechanisms of renal fibrosis is essential for developing new therapeutic targets to prevent CKD.

In addition to the two conventional factors, genes and the environment, which contribute to the etiology of diseases, increasing evidence suggests that prenatal (intrauterine) procedures have a profound effect on subsequent organ function and adult disease [5]. The ‘Developmental Origins of Health and Adult Disease’ (DOHAD) hypothesis proposes that environmental stimulation on organ development in uterus may have permanent adverse effects and increase risks of specific diseases in adult life [6–7]. Many studies have shown that intrauterine malnutrition, infection, drugs, toxins and stress are all determinants of the intrauterine environment [8–11]. The role of an adverse intrauterine environment in developmental programming can be applied to CKD in the adult [12]. Among all factors identified, low birth weight (LBW) is the most prominent factor for DOHAD. It is reported that LBW is related to the increased risk of adult-onset diseases (including renal function disorder) [13].

DBP is known as one of representative environmental endocrine-disrupting compounds (EEDs) and is ubiquitously present in a variety of consumer products [14]. Recent evidence indicates that DBP exposure in utero has an adverse influence on the male reproductive system, leading to spermatogenesis dysfunction, cryptorchidism and hypospadias [15–21]. Our previous studies showed that prenatal DBP exposure can result in hypospadias or anorectal malformations (ARMs) in male offspring [20, 22–24]. We found that DBP-induced ARMs in male offspring were accompanied by a poor general condition, LBW and significantly decreased organ/body weight ratios of the kidney [25]. Furthermore, the number of glomeruli shows a positive correlation with kidney weight [26]. Our recent finding suggests that androgen and its receptor (AR) and Fgf10/Fgfr2 may be involved in kidney abnormal development induced by DBP [27].

In this study, we used an optimized treatment regimen to show that maternal DBP exposure can induce kidney dysplasia in rat offspring on postnatal day 1 (PND1) and renal fibrosis in adult offspring. *In vitro*, DBP promoted the activation of NRK49F cells and G2/M arrest in NRK52E cells at a sublethal dose. The effect of DBP on these cell lines was linked to the generation of oxidative stress. In addition, our experimental results showed that DBP induced oxidative stress in both renal fibroblasts and tubular epithelial cells, while vitamin C ameliorated the changes caused by DBP. These results show that prenatal DBP exposure may generate oxidative stress in both renal fibroblasts and tubular epithelial cells and eventually lead to kidney dysplasia and renal fibrosis.

## RESULTS

### Maternal exposure to DBP induces kidney dysplasia in rat offspring on PND1

**Figure 1:**
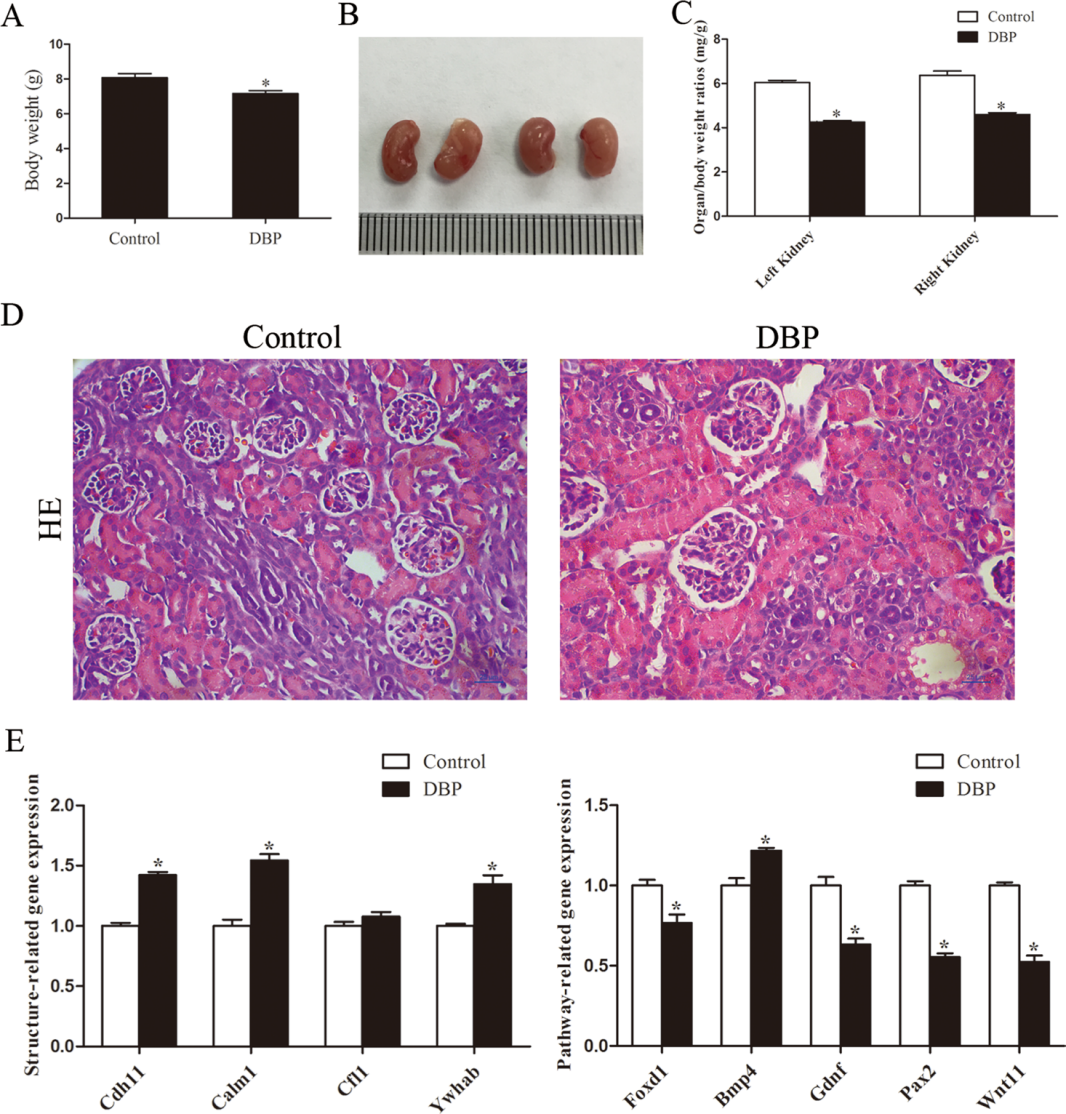
Maternal exposure to DBP induces kidney dysplasia in rat offspring on PND1 (**A**) The body weight of the rat offspring in the DBP-exposed group and DBP-unexposed controls on PND1. (**B**) Gross images of the kidneys in the DBP-exposed group and DBP-unexposed controls on PND1. (**C**) The organ/body weight ratio of the kidneys in the DBP-exposed group and the DBP-unexposed control group on PND1. (**D**) H&E stained images of kidney tissue showed differences between the control and DBP-exposed offspring on PND1. (**E**) Real-time PCR assessed the expression of genes involved in renal development (*Foxd1*, *Gdnf*, *Pax2*, *Wnt11*, *Bmp4*, *Cdh11*, *Calm1*, *Cfl1*, *Ywhab*) in the kidneys of DBP-exposed rats and DBP-unexposed controls on PND1. Data represent mean ± SD. *n* = 6 rats per group, *P < 0.05.

On PND1, the body weight (Figure 1A) and the size (Figure 1B) of the kidneys in the DBP-exposed group were significantly less than those in the DBP-unexposed controls. Compared with the DBP-unexposed control group, the organ/body weight ratio of the kidneys in the DBP-exposed group was decreased (Figure 1C). Histopathological examination of kidney tissue showed no pathological variation in control group and swelling of the glomerular tufts in the DBP-exposed group. Forkhead box D1 (Foxd1), wingless-type MMTV integration site family, member 11 (Wnt11), paired box 2 (Pax2) and glial cell derived neurotrophic factor (Gdnf) are related to renal development. The reduced mRNA expression of these genes was found in the DBP-exposed group on PND1. In addition, maternal DBP exposure led to upregulation of the mRNA expression of other genes, including bone morphogenetic protein 4 (Bmp4), cadherin 11 (Cdh11), tyrosine 3-monooxygenase/tryptophan 5-monooxygenase activation protein, beta (Ywhab) and calmodulin 1 (Calm1) (Figure 1E).

### Maternal exposure to DBP leads to renal fibrosis in adult offspring

**Figure 2:**
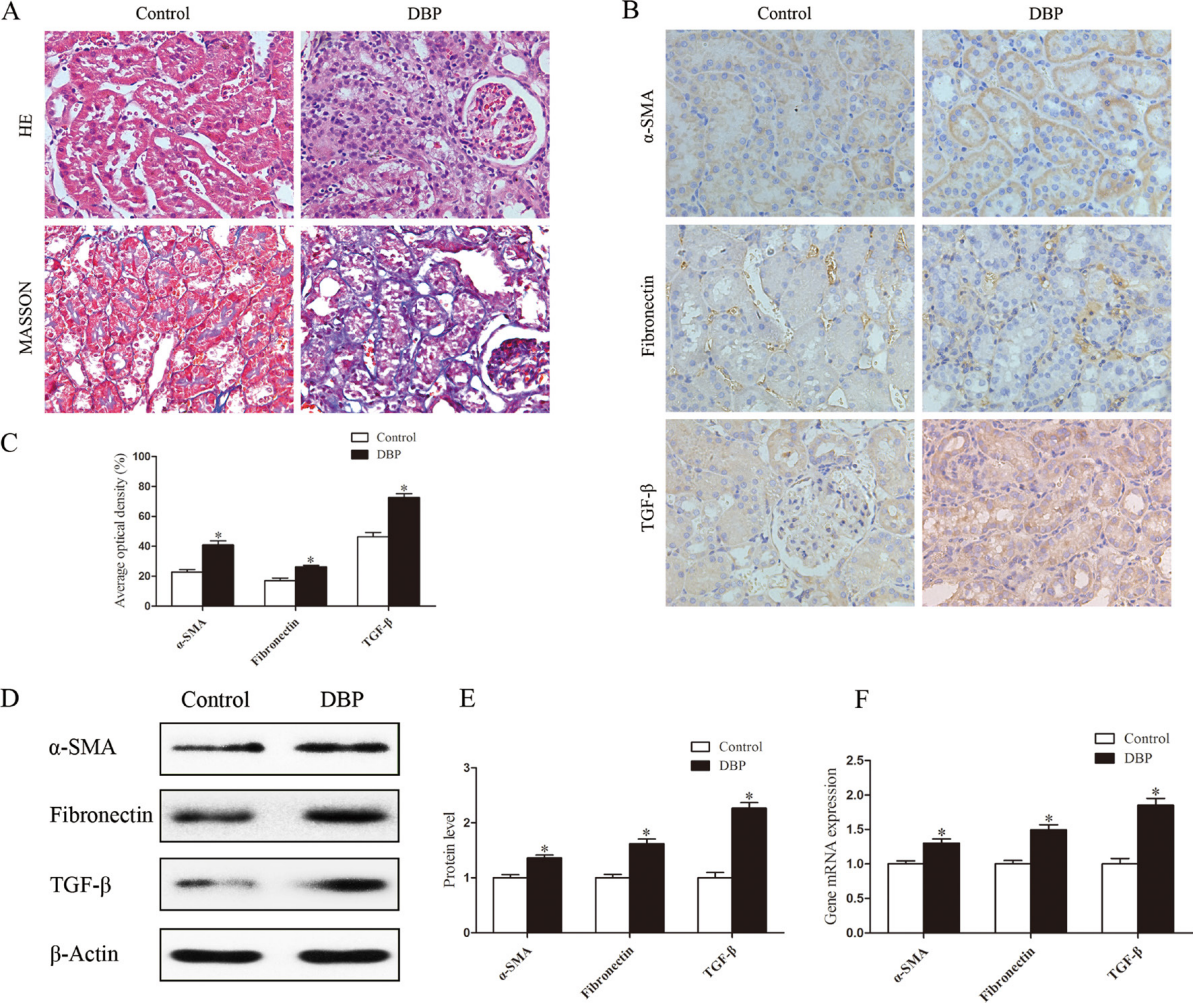
Maternal exposure to DBP leads to renal fibrosis in adult offspring (**A**) H&E and Masson trichrome stained images of kidneys in the DBP-exposed adult offspring and DBP-unexposed controls. (**B**) Represented immunohistochemical localization of α-SMA, fibronectin and TGF-β in the kidneys of DBP-exposed adult offspring and unexposed controls. (**C**) The average optical density of α-SMA, fibronectin and TGF-β in the kidneys of DBP-exposed adult offspring and unexposed controls. (**D**) Western blot showing different α-SMA, fibronectin and TGF-β protein levels of kidneys in DBP-exposed adult offspring and unexposed controls. (**E**) Densitometric quantification of α-SMA, fibronectin and TGF-β proteins in the kidneys of DBP-exposed adult offspring and unexposed controls. (**F**) Real-time PCR assessed the expression of α-SMA (*Acta*), fibronectin (*Fn*) and TGF-β (*Tgfb*) in the kidneys of DBP-exposed adult offspring and unexposed controls. Data represent mean ± SD. *n* = 6 rats per group, *P < 0.05.

Histopathological examination of kidney tissue showed that the kidneys of the DBP-exposed group developed tubular damage, with the presence of tubular atrophy, compression of tubular cells, widening of intertubular spaces and thickening of the tubular basement membrane. In addition, Masson trichrome staining showed that, compared with control rats, DBP-exposed rats exhibited significantly increased interstitial extracellular matrix accumulation (Figure 2A). Immunohistochemical staining demonstrated that the abundance of alpha-smooth muscle actin (α-SMA), fibronectin and transforming growth factor-β (TGF-β) protein was significantly higher in the kidneys of DBP-exposed adult offspring than in those of unexposed controls (Figure 2B–2C). Quantitative determinations using Western blot and real-time PCR produced similar results (Figure 2D–2F).

### DBP promotes the proliferation and activation of NRK49F cells at a sublethal dose

**Figure 3:**
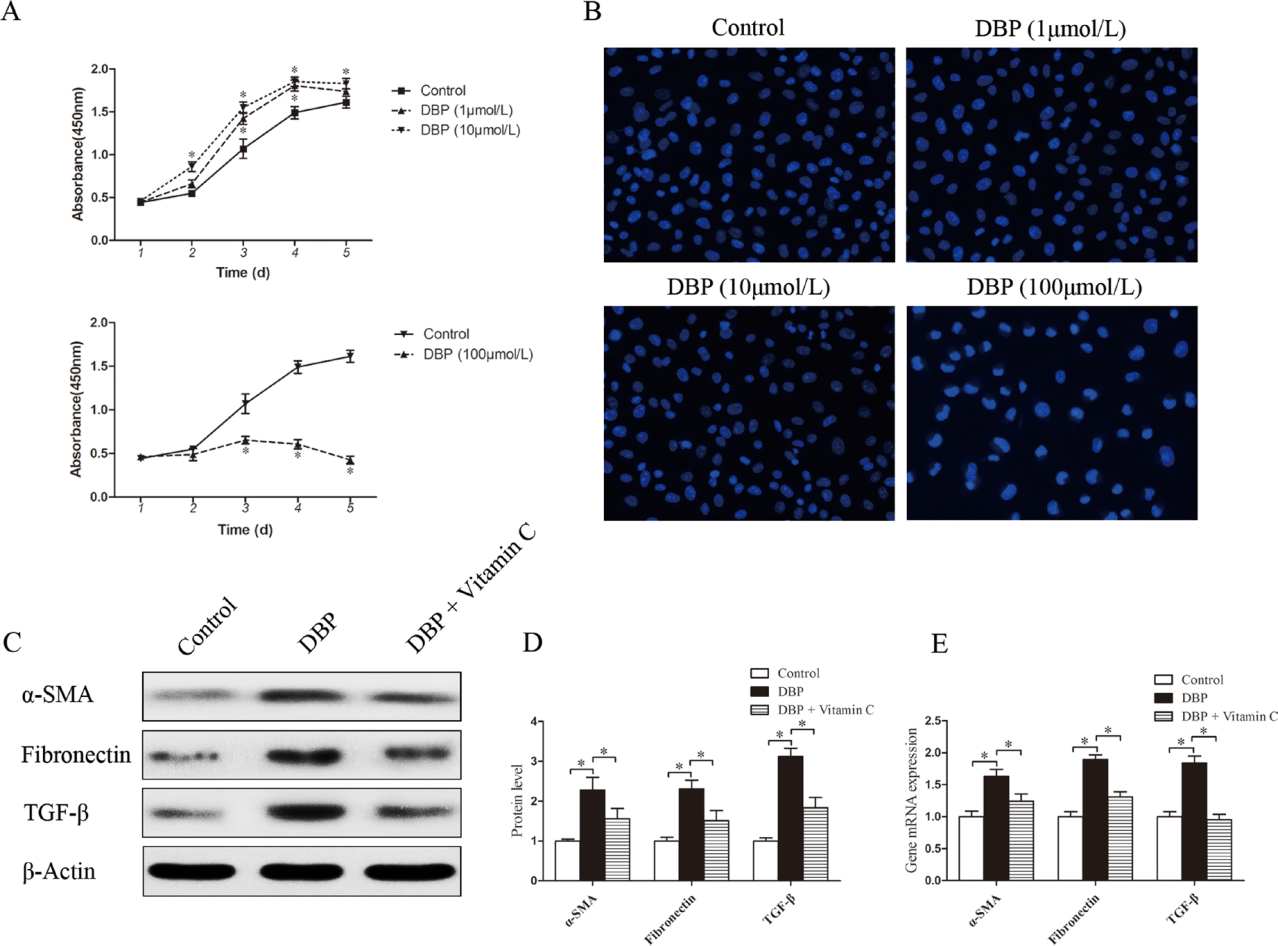
DBP promotes the proliferation and activation of NRK49F cells at a sublethal dose (**A**) CCK-8 cell proliferation assays showing different proliferation between NRK49F cells exposed to various concentrations of DBP (1, 10, 100 μmol/L). (**B**) Hoechst 33342 dye stained images of NRK49F cells exposed to various concentrations of DBP (1, 10, 100 μmol/L). (**C**) Western blot showing different α-SMA, fibronectin and TGF-β protein levels of NRK49F cells in DBP-exposed, vitamin C-exposed group and unexposed controls. (**D**) Densitometric quantification of α-SMA, fibronectin and TGF-β proteins levels of NRK49F cells in DBP-exposed, vitamin C-exposed group and unexposed controls. (**E**) Real-time PCR assessed the expression of α-SMA (*Acta*), fibronectin (*Fn*) and TGF-β (*Tgfb*) of NRK49F cells in DBP-exposed, vitamin C-exposed group and unexposed controls. Data represent mean ± SD. *n* = 6 rats per group, *P < 0.05.

To determine the effect of DBP on renal fibroblasts *in vitro*, NRK49F cells, a rat kidney interstitial fibroblast cell line, were exposed to various concentrations of DBP. At a sublethal dose (1, 10 μmol/L), DBP was capable of promoting the proliferation of NRK49F cells, whereas higher doses (100 μmol/L) caused a sharp drop in cell number (Figure 3A). To confirm the induction of apoptosis among NRK49F cells exposed to different concentrations of DBP, we stained the cells with Hoechst 33342 dye. DBP at a higher dose (100 μmol/L) induced apoptosis in NRK49F cells, whereas DBP at a sublethal dose (1, 10 μmol/L) had few effects on apoptosis in NRK49F cells (Figure 3B). Thus, we conclude that DBP promotes the proliferation of renal fibroblast NRK49F cells at a sublethal dose. In addition, in the NRK49F cells exposed to DBP at a sublethal dose (10 μmol/L), the expression levels of α-SMA, fibronectin and TGF-β were increased at both the protein and mRNA levels, compared to the control group (Figure 3C–3E).

### DBP inhibits NRK52E cell growth without apoptosis at a sublethal dose

**Figure 4:**
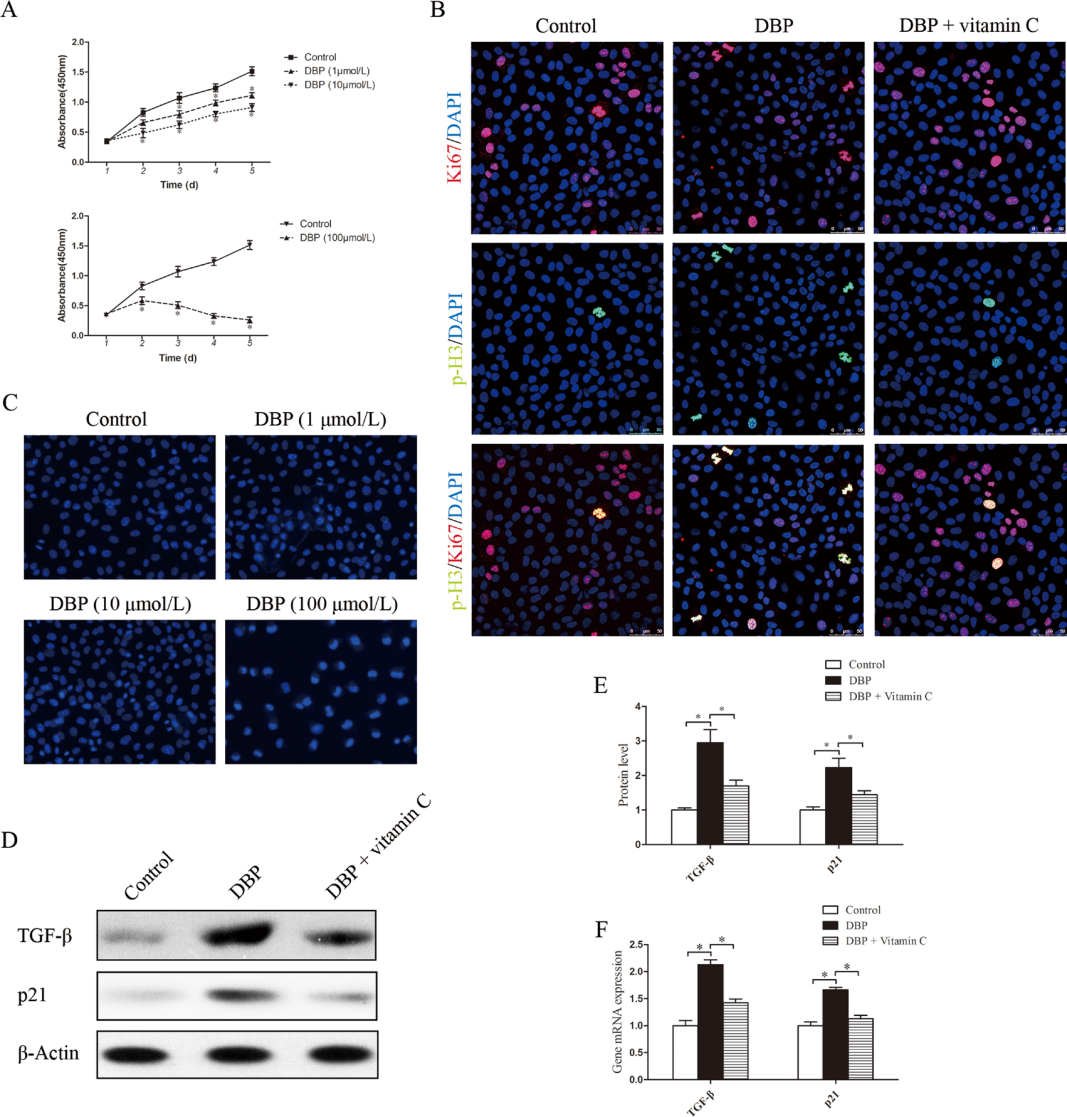
DBP inhibits NRK52E cell growth and induces G2/M arrest and the overproduction of TGF-β in NRK52E cells at a sublethal dose (**A**) CCK-8 cell proliferation assays showing different proliferation between NRK52E cells exposed to various concentrations of DBP (1, 10, 100 μmol/L). (**B**) Dual immunofluorescence of Ki67 (red) and p-H3 (green) in NRK52E cells of DBP-exposed, vitamin C-exposed group and unexposed controls. DAPI (blue) was used for nuclear staining. (**C**) Hoechst 33342 dye stained images of NRK52E cells exposed to various concentrations of DBP (1, 10, 100 μmol/L). (**D**) Western blot showing different TGF-β and p21 protein levels of NRK52E cells in DBP-exposed, vitamin C-exposed group and unexposed controls. (**E**) Densitometric quantification of TGF-β and p21 protein levels of NRK52E cells in DBP-exposed, vitamin C-exposed group and unexposed controls. (**F**) Real-time PCR assessed the expression of TGF-β (*Tgfb*) and p21(*Cdkn1a*) of NRK52E cells in DBP-exposed, vitamin C-exposed group and unexposed controls. Data represent mean ± SD. *n* = 6 rats per group, *P < 0.05.

To determine the effect of DBP on renal tubular epithelial cells *in vitro*, NRK52E cells were exposed to various concentrations of DBP. DBP at a sublethal dose (1, 10 μmol/L) had only a small effect on cell number, whereas higher doses (100 μmol/L) caused a significant decrease in cell number (Figure 4A). To test whether DBP induced apoptosis in NRK52E cells, we stained NRK52E cells with Hoechst 33342 dye. DBP at a higher dose (100 μmol/L) induced apoptosis of NRK52E cells, whereas DBP at a sublethal dose (1, 10 μmol/L) had few effects on apoptosis in NRK52E cells (Figure 4C). Thus, we conclude that DBP inhibits the proliferation of NRK52E cells without cell death at a sublethal dose.

### DBP induces G2/M arrest and the overproduction of TGF-β in NRK52E cells

With the Ki67/ the phosphorylation of histone H3 at Ser10 (p-H3) double staining, we evaluated whether NRK52E cells arrest at the G2/M phase. Among all proliferative NRK52E cells (Ki67-positive), the percentage of NRK52E cells exposed to DBP in the G2/M phase (p-H3 positive) was increased significantly, compared with the percentage of the DBP-unexposed controls (Figure 4B). Furthermore, Western blot analysis and real-time PCR showed that the expression of TGF-β was significantly higher in the NRK52E exposed to DBP at a sublethal dose (Figure 4D–4F). Positive regulation of the cell cycle is mediated by the cyclin-dependent kinases. p21 is one of the CKD inhibitors and belongs to the Cip/Kip family [28]. The expression of p21 protein was investigated in NRK52E cells by Western blot analysis, and the results showed significantly increased expression of p21 in DBP-exposed NRK52E cells. Quantitative determination of the mRNA level by real-time PCR showed similar results (Figure 4D–4F).

### DBP generates oxidative stress in NRK49F and NRK52E cells

**Figure 5:**
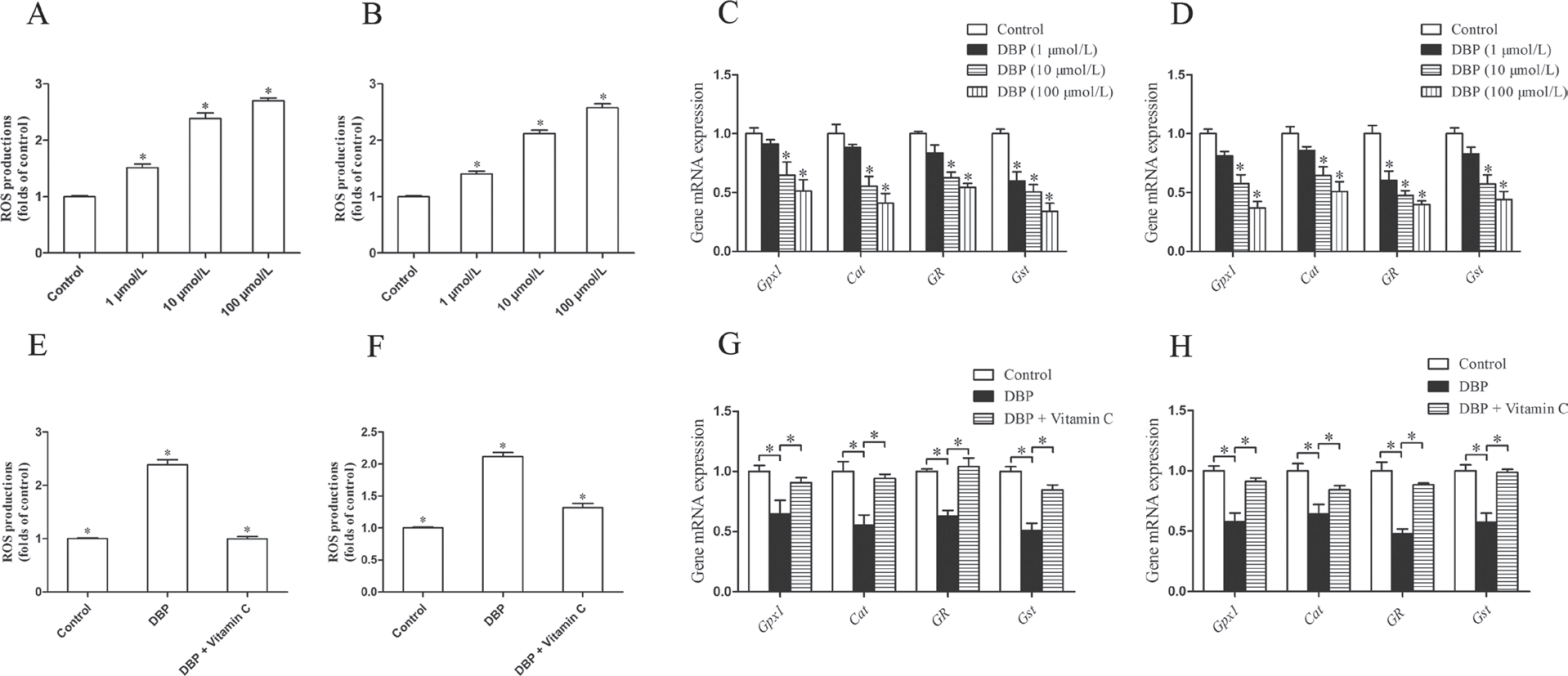
DBP generates oxidative stress and reduces the expression of the gene responsible for the prevention of oxidative activity in NRK49F and NRK52E cells (**A**) Measurement of intracellular ROS in NRK49F cells by DCFH-DA dye showed the quantity of ROS increased significantly after treatment with various concentrations of DBP (1, 10, 100 μmol/L). (**B**) Measurement of intracellular ROS in NRK52E cells by DCFH-DA dye showed the quantity of ROS increased significantly after treatment with various concentrations of DBP (1, 10, 100 μmol/L). (**C**) Real-time PCR assessed the expression of antioxidant genes (*Gpx1, Cat, GR, Gst*) of NRK49F cells exposed to various concentrations of DBP (1, 10, 100 μmol/L). (**D**) Real-time PCR assessed the expression of antioxidant genes (*Gpx1, Cat, GR, Gst*) of NRK52E cells exposed to various concentrations of DBP (1, 10, 100 μmol/L). (**E**) Measurement of intracellular ROS by DCFH-DA dye in NRK49F cells in DBP-exposed, vitamin C-exposed group and unexposed controls. (**F**) Measurement of intracellular ROS by DCFH-DA dye in NRK52E cells in DBP-exposed, vitamin C-exposed group and unexposed controls. (**G**) Real-time PCR assessed the expression of antioxidant genes (*Gpx1, Cat, GR, Gst*) of NRK49F cells in DBP-exposed, vitamin C-exposed group and unexposed controls. (**H**) Real-time PCR assessed the expression of antioxidant genes (*Gpx1, Cat, GR, Gst*) of NRK52E cells in DBP-exposed, vitamin C-exposed group and unexposed controls. Data represent mean ± SD. *n* = 6 rats per group, *P < 0.05.

NRK49F and NRK52E cells were incubated with DCFH-DA to measure the intracellular reactive oxygen species (ROS). The quantity of ROS in these cells increased significantly after treatment with DBP (*P <* 0.05) (Figure 5A–5B). The result suggested that DBP induced the generation of ROS in NRK49F and NRK52E cells, which was obvious in higher concentration of DBP.

### DBP reduces the expression of the gene responsible for the prevention of oxidative activity in NRK49F and NRK52E cells

The impact of DBP on the antioxidant activity of genes in NRK49F and NRK52E cells was analyzed by real-time PCR. We found that DBP significantly reduced the expression of antioxidant genes such as glutathione peroxidase 1 (*Gpx1*), catalase (*Cat*), glutathione reductase (*GR*) and glutathione-s-transferase (*Gst*). Expression of these antioxidant genes was decreased with increasing doses of DBP (Figure 5C–5D).

### The effect of DBP on NRK49F and NRK52E cells can be reversed by vitamin C

NRK49F and NRK52E cells were pretreated with vitamin C, an important water-soluble antioxidant. The results showed that vitamin C could protect these cells against the oxidative stress induced by DBP. The quantity of ROS decreased significantly in NRK49F and NRK52E cells co-incubated with DBP and vitamin C compared to that in cells treated with DBP alone (Figure 5E–5F). The expression of antioxidant genes was also increased in the these cells co-incubated with DBP and vitamin C (Figure 5G–5H). Furthermore, vitamin C almost completely blocked the G2/M arrest induced by DBP (Figure 4B). The expression of α-SMA, fibronectin and TGF-β in NRK49F cells decreased to some degree with vitamin C pretreatment (Figure 3C–3E). In addition, the overproduction of TGF-β in NRK52E cells induced by DBP was reversed by treatment with vitamin C (Figure 4D–4F).

## DISCUSSION

The present study demonstrates that maternal DBP exposure can induce kidney dysplasia in offspring on PND1 and renal fibrosis in adult offspring. *In vitro*, DBP promoted the activation of NRK49F cells and G2/M arrest in NRK52E cells at a sublethal dose. The effect of DBP on these cell lines was linked to the generation of oxidative stress, and treatment with vitamin C could ameliorate the changes caused by DBP.

DBP is an estrogen-like chemical that is ubiquitously present in a variety of consumer products and can also be released into the environment. Most studies indicate that DBP has an adverse influence on the reproductive system. Our previous studies have shown that maternal exposure to DBP can induce decreased organ/body weight ratios in the kidney [25]. In the present study, we detected swelling of the glomerular tufts in the kidney of the DBP-exposed group on PND1 by histological examination. *Foxd1*, *Gdnf*, *Pax2* and *Wnt1* are related to branching of ureteric bud and inducing nephron [29]. In the study, we found maternal DBP exposure led to reduced *Foxd1*, *Gdnf*, *Pax2* and *Wnt1* mRNA expressions. Bmp4 can inhibit Gdnf signaling, which is consistent with our finding in the study. The upregulation of the mRNA expression of structure-related genes *Cdh11*, *Calm1* and *Ywhab* in rat offspring on PND1 suggests the renal development is incomplete [29]. Maternal exposure to DBP also led to renal fibrosis in adult offspring. Histopathological examination of the kidney tissue showed that the kidneys of the DBP-exposed group developed tubular damage and increases in the interstitial space. In addition to interstitial extracellular matrix accumulation, proteins such as α-SMA, fibronectin, and TGF-β were significantly increased in the DBP-exposed group.

A recent study showed that the activities of antioxidant enzymes and total antioxidant capacity in serum decreased significantly after treatment with DBP [30]. Another ATM substrate Chk2-interacting Zn(2+)-finger knockout mouse study comfirmed that oxidative damage can lead to severe organ development defects [31]. These results indicate that oxidative stress could be one of the mechanisms responsible for the development DBP-induced kidney dysplasia. It has been shown that BPA, another EED, can down-regulate the antioxidant gene expression and induce the production of ROS [32]. The present study demonstrates that in NRK49F and NRK52E cells, the quantity of ROS increased and the expression of antioxidant genes (*Gpx1*, *Cat*, *Gst*, and *GR*) decreased significantly after treatment with DBP.

Aberrant activation of fibroblasts to myofibroblasts lead to relentless deposition of extracellular matrix, including α-SMA and fibronectin. This process is closely related to oxidative stress. TGF-β1 can activate fibroblasts to myofibroblasts and promote the development of renal fibrosis via Smad-dependent and Smad-independent signaling pathways. [3, 33]. In our study, the expression levels of α-SMA, fibronectin and TGF-β were increased in NRK49F cells exposed to DBP at a sublethal dose, which suggests that the activation of renal fibroblasts by DBP leads to renal fibrosis.

In our study, DBP also induced G2/M arrest and the overproduction of TGF-β in NRK52E cells. DNA damage caused by oxidative stress is known to lead to G2/M arrest [34]. A recent study revealed the relationship between tubular epithelial cells arrested in the G2/M phase and progression of renal fibrosis. The cell cycle dysregulation in tubular epithelial cells was correlated with overproduction of TGF-β1 and led to renal fibrosis, while reversal of G2/M arrest reduced the production of TGF-β1 and attenuated the progression of renal fibrosis [35]. Indeed, tubular epithelial cells arrested in the G2/M phase play a crucial role in the pathogenesis of TIF.

Vitamin C is one of the most important water-soluble antioxidants. Studies have shown that vitamin C has antioxidant capacities and protective properties against oxidative stress injury caused by ROS [36–37]. Vitamin C has been reported to have a protective effect against renal injury by reducing the oxidative stress caused by hind limb ischemia-reperfusion (I/R) [38]. In our study, the effect of DBP on NRK49F and NRK52E cells could be reversed by treatment with vitamin C. Compared to cells treated with DBP alone, in cells co-incubated with DBP and vitamin C, the quantity of ROS decreased and the expression of antioxidant genes increased significantly. Furthermore, vitamin C almost completely blocked the G2/M arrest induced by DBP, and the expression of α-SMA, fibronectin and TGF-β in NRK49F cells decreased to some degree when the cells were pretreated with vitamin C. In addition, TGF-β overproduction in NRK52E cells induced by DBP could be reversed by vitamin C. Together, these findings suggest that the oxidative stress induced by DBP can activate renal fibroblasts, increase the G2/M phase arrest of tubular epithelial cells, and promote progression of renal fibrosis. However, vitamin C can protect tubular epithelial cells and renal fibroblasts from oxidative stress caused by DBP.

Our studies use an optimized treatment regimen of DBP and are the first to show that maternal exposure to DBP can induce kidney dysplasia in offspring on PND1 and renal fibrosis in adult offspring. *In vitro*, DBP could promote the activation of NRK49F cells and G2/M arrest in NRK52E cells at a sublethal dose. The effect of DBP on these cell lines was linked to the generation of oxidative stress. In addition, we determined experimentally that DBP induced oxidative stress in both renal fibroblasts and tubular epithelial cells, whereas vitamin C ameliorated the changes caused by DBP. These results show that maternal exposure to DBP may generate oxidative stress in both renal fibroblasts and tubular epithelial cells, leading to kidney dysplasia and renal fibrosis. According to the DOHAD hypothesis, maternal exposure to DBP may damage the development of the kidneys *in utero* and have permanent deleterious effects that increase the risk of CKD (renal fibrosis) during adulthood. Thus, it is important to avoid exposure to DBP and other EEDs during pregnancy to decrease the risk of renal fibrosis. In addition, treatment with vitamin C during pregnancy may reduce DBP-induced damage to the developing kidneys.

## MATERIALS AND METHODS

### Animals

Sprague-Dawley rats were supplied by Shanghai Laboratory Animal Center. Ethical approval for animal experiments was obtained from the National Institute of Health's Guide for the Care and Use of Laboratory Animals. The method of mating was performed as previously described [23–24, 27]. Twenty successfully mated female rats were distributed into control and DBP-exposed groups randomly. The exposure period and dosage levels of DBP were based on our previous study which effectively induce kidney dysplasia in offspring [27]. Six rats were selected randomly from the DBP-exposed offspring and controls on PND1 and at 18 months, respectively. After measurement of the organ/body weight ratios, the kidneys were collected for subsequent experiments.

### Cells and exposure conditions

NRK49F cells and NRK52E cells were obtained from the American Type Culture Collection (ATCC, Manassas, VA). The cells were maintained in Dulbecco's modified Eagle's medium (DMEM) (HyClone, Logan, UT) supplemented with 10% fetal bovine serum (FBS) (Gibco, Foster City, CA) and 1% penicillin/streptomycin (HyClone), and the cells were cultured at 37°C in a 5% CO_2_ environment. DBP exposure solutions were prepared by dissolving DBP in DMSO and adding this solution to the medium up to concentrations of 1, 10, or 100 μmol/L. The concentration of DBP in solvent (DMSO) did not exceed 0.5% (v/v). Vitamin C [(+)-sodium L-ascorbate] (Sigma Chemical Co., St. Louis, MO, USA) was dissolved in sterile deionized water. The antioxidant dose of vitamin C was determined to be 50 μM. Accordingly, this concentration of vitamin C was added along with DBP to the culture medium. The solutions of DBP and DBP + vitamin C were sterilized by passage through a 0.22-μm filter (Millipore Ireland, Cork, Ireland) before use.

### Histology

The kidney tissues were fixed with formalin. Paraffin-embedded 4-mm kidney sections were stained with hematoxylin-eosin (H&E) and Masson's trichrome (Sigma-Aldrich).

### Western blot

Western blot was performed as previously described [23–24, 27]. The primary antibodies against α-SMA (1:200, Sigma, MO, USA), fibronectin (1:1000, Abcam, Cambridge, UK), TGF-β (1:1000, Cell Signaling Technology, MA, USA), or p21 (1:200, Santa Cruz Biotechnology, CA, USA) were used in the study.

### Immunohistochemistry

Immunohistochemistry was performed as previously described [27]. The primary antibodies against α-SMA (1:200, Sigma, MO, USA), fibronectin (1:500, Abcam, Cambridge, UK), and TGF-β (1:500, Cell Signaling Technology, MA, USA) were used in the study. After incubation with the primary antibodies, the sections were incubated with secondary antibody (D-3004, Long Island Biotech, Shanghai, China).

### Hoechst staining

After treatment with DBP (1, 10, or 100 μmol/L) for 24 h, the cells were stained with 0.5 μg/ml Hoechst 33342 (Life Technologies, USA) for 25 min at 37°C. The concentration of DBP in solvent (DMSO) did not exceed 0.5% (v/v). Finally, the cells were observed under a fluorescence microscope (Leica, Wetziar, Germany).

### Cell Counting Kit-8 (CCK-8)

Cell proliferation assays were performed using Cell Counting Kit-8 (CCK-8, Dojindo, Kumamoto, Japan) reagents. CCK-8 experiments were carried out for 5 consecutive days by adding 40 μl of CCK-8 reagent and 400 μl of PBS to each well, with incubation at 37°C for 2 h. Viable cells were evaluated by absorbance measurements at 450 nm.

### Real-time polymerase chain reaction (real-time PCR)

Total RNA of kidney tissues and cells was extracted using Trizol reagent (Invitrogen, Carlsbad, CA, USA). Reverse transcript PCR for mRNA was carried out using Superscript II reverse transcriptase and random hexamers (Invitrogen, Carlsbad, CA, USA). 0.8μg mRNA was used for the synthesis of 20μl cDNA. Real-time PCR was performed on an ABI PRISM 7300 Sequence Detection System using SYBR Green PCR (Applied Biosystems). Primer pairs detailed in Table 1.

**Table 1:** Oligonucleotide primers used in this study

Gene	Forward primer	Reverse primer
*Foxd1*	TCACTCCTCCCGCGCCCTTC	GCTCCGCTCCGCTACTTGGC
*Gdnf*	GGTGTTGCTCCACACCGCGT	GGTGGCCGAGGGAGTGGTCT
*Pax2*	CCCAAGGTGGCAACGCCCAA	CGGCGTTGGGTGGAAAGGCT
*Wnt11*	TCCATCGAGCTCGCCCCCAA	TGGCCCCCATGAGGAGACCG
*Bmp4*	ACCACCTCAACTCAACCAATC	TCCACCACCATCTCCTGATAA
*Cdh11*	GCCGCCGCCGACTTGTGAAT	ATTTCTGGGGCCGTTGCGGG
*Calm1*	GAATGGCACCATTGACTTCC	GTAGCCATTGCCATCCTTGT
*Cfl1*	TTCTGGTAGGAGATGTGGGG	ACCAGGTCCTCCTTCTTGCT
*Ywhac*	CCGGTATCTTGCTGAAGTCG	GGATCGGATGTGTAGGTTGC
*Acta*	CGATAGAACACGGCATCATC	CATCAGGCAGTTCGTAGCTC
*Tgfb*	TGAGTGGCTGTCTTTTGACG	TGGTTGTAGAGGGCAAGGAC
*Fn*	GGGAAGAAAAGGAGCCCAGG	GGAAAAGTCCTGAGGTGGGG
*Cdkn1a*	CGAGAACGGTGGAACTTTGAC	GAAATCTGTTAGGCTGGTCTGC
*Gpx1*	GGGCAAAGAAGATTCCAGGTT	AGAGCGGGTGAGCCTTCT
*Cat*	AGGTGACACTATAGAATAGTG GTTTTCACCGACGAGAT	GTACGACTCACTATAGGGACA CGAGGTCCCAGTTACCAT
*GR*	TTCTGGAACTCGTCCACTAGG	CCATGTGGTTACTGCACTACTTCC
*Gst*	GCCTTCTACCCGAAGACACCTT	GTCAGCCTGTTCCCTACA
*Gapdh*	ACAGCAACAGGGTGGTGGAC	TTTGAGGGTGCAGCGAACTT

Abbreviations: forkhead box D1 (Foxd1), glial cell derived neurotrophic factor (Gdnf), paired box 2 (Pax2) and wingless-type MMTV integration site family, member 11 (Wnt11), bone morphogenetic protein 4 (Bmp4), cadherin 11 (Cdh11), calmodulin 1 (Calm1), cofilin 1 (Cfl1), tyrosine 3-monooxygenase/tryptophan 5-monooxygenase activation protein, beta (Ywhab), alpha-smooth muscle actin (Acta), transforming growth factor, beta (Tgfb), fibronectin (Fn), cyclin-dependent kinase inhibitor 1A (Cdkn1a), glutathione peroxidase 1 (Gpx1), catalase (Cat), glutathione reductase (GR) and glutathione-s-transferase (Gst).

### Immunofluorescence staining

Paraffin-fixed cells were permeabilized and incubated with a primary antibody overnight at 4°C, followed by incubated with secondary antibody conjugated to Alexa Fluor 488 or 588 (Molecular Probes, Inc., Eugene, OR, USA). The slides were then counterstained with 4′, 6-diamidino-2- phenylindole (DAPI) to visualize the nuclei. Images were obtained using confocal microscopy (TCS SP8; Leica, Wetzlar, Germany). The primary antibodies used in this study included anti-p-H3 (Ser10) (1:1000, Abcam, Cambridge, UK) and anti-Ki67 (1:1000, Abcam, Cambridge, UK).

### Measurement of intracellular ROS

Following treatment with final concentrations of DBP (1, 10, or 100 μmol/L) for 24 h, cells were incubated with DCFH-DA dye (50 μmol/L, final concentration) in medium for 30 min in the dark. After rinsing twice with PBS solution, lysis buffer was added to the dishes to disrupt the cell membranes. Cell suspensions were transferred to 96-well plates, and the fluorescence was read at an excitation wavelength of 490 nm and emission of 520 nm.

### Statistical analysis

Differences were analyzed using Mann-Whitney *U* test or an independent-samples *t-test*. Stata 7.0 software (Stata Corporation, College Station, USA) was used to analyze all data. Statistical significance was set at *P <* 0.05.

## Abbreviations

DBP: di-n-butyl phthalate
TIF: tubulointerstitial fibrosis
PND1: postnatal day 1
CKD: chronic kidney disease
DOHAD: Developmental Origins of Health and Adult Disease
LBW: low birth weight
EEDs: Environmental endocrine-disrupting compounds
ARMs: anorectal malformations
AR: androgen receptor
Foxd1: forkhead box D1
Gdnf: glial cell derived neurotrophic factor
Pax2: paired box 2
Wnt11: wingless-type MMTV integration site family, member 11
Bmp4: bone morphogenetic protein 4
Cdh11: cadherin 11
Calm1: calmodulin 1
Ywhab: tyrosine 3-monooxygenase/tryptophan 5-monooxygenase activation protein, beta
α-SMA: alpha-smooth muscle actin
TGF-β: transforming growth factor-β
p3H: phosphorylation of histone H3 at Ser10
ROS: reactive oxygen species
Gpx1: glutathione peroxidase 1
Cat: catalase
RG: glutathione reductase
Gst: glutathione-s-transferase
GD: gestation day
DMEM: Dulbecco's modified Eagle's medium
FBS: fetal bovine serum
H&E: hematoxylin-eosin
CCK-8: Cell Counting Kit-8
real-time PCR: Real-time polymerase chain reaction
DAPI: 49, 6-diamidino-2- phenylindole
SD: standard deviation
